# Retraction: Human swimming posture recognition combining improved 3D convolutional network and attention residual network

**DOI:** 10.1371/journal.pone.0351261

**Published:** 2026-06-23

**Authors:** The PLOS One Editors

The *PLOS One* Editors retract this article [1] because it was identified as one of a series of submissions for which we have concerns about potential manipulation of the publication process. These concerns call into question the validity and provenance of the reported results. We regret that the issues were not identified prior to the article’s publication.

All authors either did not respond directly or could not be reached.

## References

[1] Li M, Ning C. RETRACTED: Human swimming posture recognition combining improved 3D convolutional network and attention residual network. PLoS One. 2025;20(12):e0337577. doi: 10.1371/journal.pone.0337577 41329706 PMC12671778

